# Arterial stiffness and shortened QTc interval are associated with androgen and ACTH levels in classic congenital adrenal hyperplasia

**DOI:** 10.3389/fendo.2025.1660114

**Published:** 2025-08-22

**Authors:** Lorenzo Campioni, Maria Chiara Di Carlo, Chiara Sola, Beatrice Bergoglio, Martina Bollati, Giulia Montesano, Federico Ponzetto, Chiara Lopez, Giovanna Motta, Mirko Parasiliti-Caprino, Roberta Giordano

**Affiliations:** ^1^ Endocrinology, Diabetes and Metabolism, City of Health and Science University Hospital, Turin, Italy; ^2^ Department of Medical Sciences, University of Turin, Turin, Italy; ^3^ Clinical Biochemistry Laboratory, City of Health and Science University Hospital, Turin, Italy; ^4^ Department of Biological and Clinical Sciences, University of Turin, Turin, Italy

**Keywords:** cardiovascular risk, congenital adrenal hyperplasia, 21-hydroxylase deficiency, arterial stiffness, androgens, ACTH, QTc

## Abstract

**Context:**

Cardiometabolic complications are increasingly recognized in congenital adrenal hyperplasia (CAH) due to 21β-hydroxylase deficiency, but adult data remain limited.

**Objective:**

To evaluate cardiovascular and metabolic alterations in adult patients with classic CAH under glucocorticoid treatment, compared to matched controls.

**Methods:**

A cross-sectional study was conducted on adults with classic CAH and sex- and BMI-matched controls. Cardiovascular and metabolic variables and parameters were collected in all patients.

**Results:**

The study enrolled 32 CAH patients and 73 controls. In univariate analyses, CAH patients showed significantly shorter QTc intervals (p=0.004), longer QRS duration and shorter RR intervals, in comparison with controls. Even in presence of a more favorable hypertensive (lower diastolic blood pressure and higher heart rate variability) and metabolic profile (lower fasting glucose, LDL cholesterol, triglycerides, and higher HDL), CAH patients had higher Ambulatory Arterial Stiffness Index (AASI) (p=0.006). Multivariate regression confirmed the association between CAH and both increased AASI (EC 1.131, p<0.001) and shortened QTc (EC 0.977, p=0.039), adjusting for all potential confounders. Within the CAH group, 17-hydroxyprogesterone was positively associated with AASI (EC 1.001, p=0.049), while ACTH (EC 0.999, p=0.021) was inversely associated with QTc, after correction for all clinical confounders. Propensity score-matched analysis with 1:2 matching ratio, based on the same regression models, confirmed that CAH diagnosis was associated with AASI (p<0.001) and QTc (p=0.004).

**Conclusions:**

Adults with classic CAH show increased arterial stiffness and altered cardiac repolarization, likely linked to chronic hormonal imbalance. These findings underscore the need for cardiovascular monitoring in long-term CAH management.

## Introduction

Congenital adrenal hyperplasia (CAH) encompasses a group of rare autosomal recessive disorders characterized by impaired cortisol synthesis and hyperandrogenism in classic form. The most common type of CAH, 21β-hydroxylase deficiency, accounts for over 90% of CAH cases and includes both the simple virilizing and salt-wasting forms (1), with a variety of clinical manifestations depending on the entity of the enzyme deficits. According to national registries, the estimated worldwide incidence ranges from 1:14,000 to 1:18,000 births. However, the prevalence is believed to be even higher in small, genetically isolated populations where consanguineous marriages are common (2).

Classic CAH due to 21β-hydroxylase deficiency is a complex disorder requiring a multidisciplinary approach to manage its comorbidities, particularly metabolic and cardiovascular complications, highlighting the importance of long-term follow-up. Moreover, there is evidence of a reduced fertility due to hyperandrogenism as well as to an increased incidence of testicular adrenal rest tumors (TARTs) in males (3, 4) and polycystic ovarian morphology (PCOM) in females (5).

Current treatment strategies for classic CAH remain suboptimal, as available glucocorticoids (GCs) fail to effectively suppress androgen hypersecretion and mimic endogenous cortisol secretion (6). Over the past decade, studies have shown that GCs dosage is often supraphysiological, increasing the risk of metabolic and cardiovascular complications and leading to higher morbidity rates (7). Recent findings suggest that modified-release formulations of hydrocortisone may help improve patients’ quality of life while reducing morbidity (8). However, till now, cardiovascular and metabolic risk in CAH patients has primarily been evaluated through small-scale retrospective studies, often yielding heterogeneous results (9, 10).

Therefore, giving the rarity of the disease, the aim of the present study was to assess the cardiovascular and metabolic risk in adult patients with classic CAH due to 21β-hydroxylase deficiency undergoing treatment with GCs formulations in comparison with a cohort of matched patients adding statistical correction to avoid potential sources of bias.

## Materials and methods

A cross-sectional study was conducted on 1) adult patients with CAH due to 21β-hydroxylase deficiency diagnosed in the last 10 years, and 2) a control group, matched for sex and BMI. All participants were recruited from the gynecological endocrinology, andrological and adrenal diseases outpatient clinic of the Division of Endocrinology, Diabetes, and Metabolism of the City of Health and Science University Hospital of Turin.

Inclusion criteria required the ability to provide informed consent and a diagnosis of classic CAH according to international guidelines, confirmed through genetic testing. Patients were excluded if they suffered from other endocrine disorders (e.g., Addison’s disease, Cushing’s syndrome, pheochromocytoma/paraganglioma, adrenal-secreting adenoma), were pregnant or breastfeeding, used medications affecting hormone levels (e.g., corticosteroids for other indications), or had pre-existing cardiovascular diseases (e.g., heart attack, stroke).

Controls were selected from a group of patients undergoing urine metanephrine analysis, in whom the suspicion of pheochromocytoma/paraganglioma was ruled out.

The study was approved by the hospital’s Ethics Committee in accordance with the principles of the Declaration of Helsinki. All participants provided informed consent. Data collection was fully anonymized using a coding system to ensure privacy and confidentiality, with each participant assigned a unique identification code for anonymity.

### Data collection

Anthropometric, biochemical, and instrumental evaluations were performed for each patient, including comorbidity assessment, with data updated at the last follow-up. According to WHO criteria (11), patients were classified as overweight (BMI 25–29.9 kg/m²), class I (30–34.9), class II (35–39.9), or class III obesity (≥40). Biochemical parameters included blood glucose, lipid panel, creatinine, and eGFR (CKD-EPI formula). Based on ADA guidelines (12), patients were categorized as having impaired fasting glucose (100–125 mg/dL), impaired glucose tolerance (2-h post-OGTT 140–199 mg/dL), or diabetes mellitus (random glucose >200 mg/dL, fasting ≥126 mg/dL, OGTT >200 mg/dL, or HbA1c ≥48 mmol/mol). Dyslipidemia was defined per ESC guidelines (13): LDL ≥116 mg/dL (low risk), ≥100 (moderate), ≥70 (high), or ≥55 (very high risk); HDL <40 mg/dL (males) or <50 mg/dL (females); triglycerides ≥150 mg/dL. Electrocardiography (ECG) was also performed, with assessments including heart rate (HR), PQ and RR intervals, and the corrected QT interval (QTc) using Bazett’s formula. PQ and RR intervals were analyzed to evaluate cardiac conduction, while QTc was examined for potential ventricular repolarization abnormalities.

### Blood pressure measurements

Office blood pressure (BP) values were collected according to guidelines (14). BP control was defined as an average office BP <140/90 mmHg. All patients underwent 24-hour ambulatory blood pressure monitoring (ABPM) using an automated, noninvasive oscillometric device (TM-2430; Intermed S.r.l., Milan, Italy). Measurements were taken every 15 minutes during the daytime and every 20 minutes at night. Valid ABPM recordings required >80% successful measurements.

Controlled ambulatory BP was defined based on current guidelines (14). Heart rate variability (HRV) was calculated as the standard deviation of daytime, nighttime, and 24-hour heart rate (HR) from ABPM data. The nocturnal BP profile was categorized as: a) reverse dipping: nighttime BP higher than daytime BP, b) reduced dipping: nighttime BP reduction of 0-10%, c) normal dipping: nighttime BP reduction of 10-20% and d) extreme dipping: nighttime BP reduction >20%.

The ambulatory arterial stiffness index (AASI) was determined using a proposed formula (15).

### Statistical analysis

Continuous variables were reported as median and 25th–75th percentiles due to their non-normal distribution (as assessed by the Shapiro-Wilk test), while categorical variables were expressed as absolute and relative frequencies (%). Comparisons between CAH patients and controls were performed using the Mann–Whitney U test for continuous variables and Fisher’s exact or Chi-square test for categorical variables, as appropriate.

Multiple linear regression models were used to investigate associations between clinical and biochemical variables and cardiovascular outcomes (AASI and QTc interval). Two models were constructed: one including the whole population and another restricted to the CAH group to explore the influence of specific hormonal variables. Despite the non-normal distribution of several independent variables, linear regression analysis was considered appropriate, as the key assumptions relate to the distribution of residuals rather than of raw data. Visual inspection of diagnostic plots confirmed the approximate normality, linearity, and homoscedasticity of residuals in all models.

To further minimize selection bias, propensity score matching (PSM) was applied using the same covariates included in the regression models, with a 1:2 matching ratio (CAH:controls). Matching balance was evaluated by comparing baseline characteristics before and after matching.

*A priori* power analysis was performed to determine the required sample size to detect a clinically meaningful difference in Ambulatory Arterial Stiffness Index (AASI) between groups. Assuming a two-tailed α of 0.05, a power of 80%, and a moderate effect size (Cohen’s d = 0.6), a minimum of 30 CAH patients and 69 controls was estimated to be necessary. Our final sample (32 CAH and 73 controls) exceeds this threshold, confirming adequate statistical power to test the primary hypothesis.

All statistical tests were two-sided, and a p-value <0.05 was considered statistically significant. Analyses were conducted using STATA version 18.0 (StataCorp, College Station, TX, USA).

## Results

**Figure 1:**
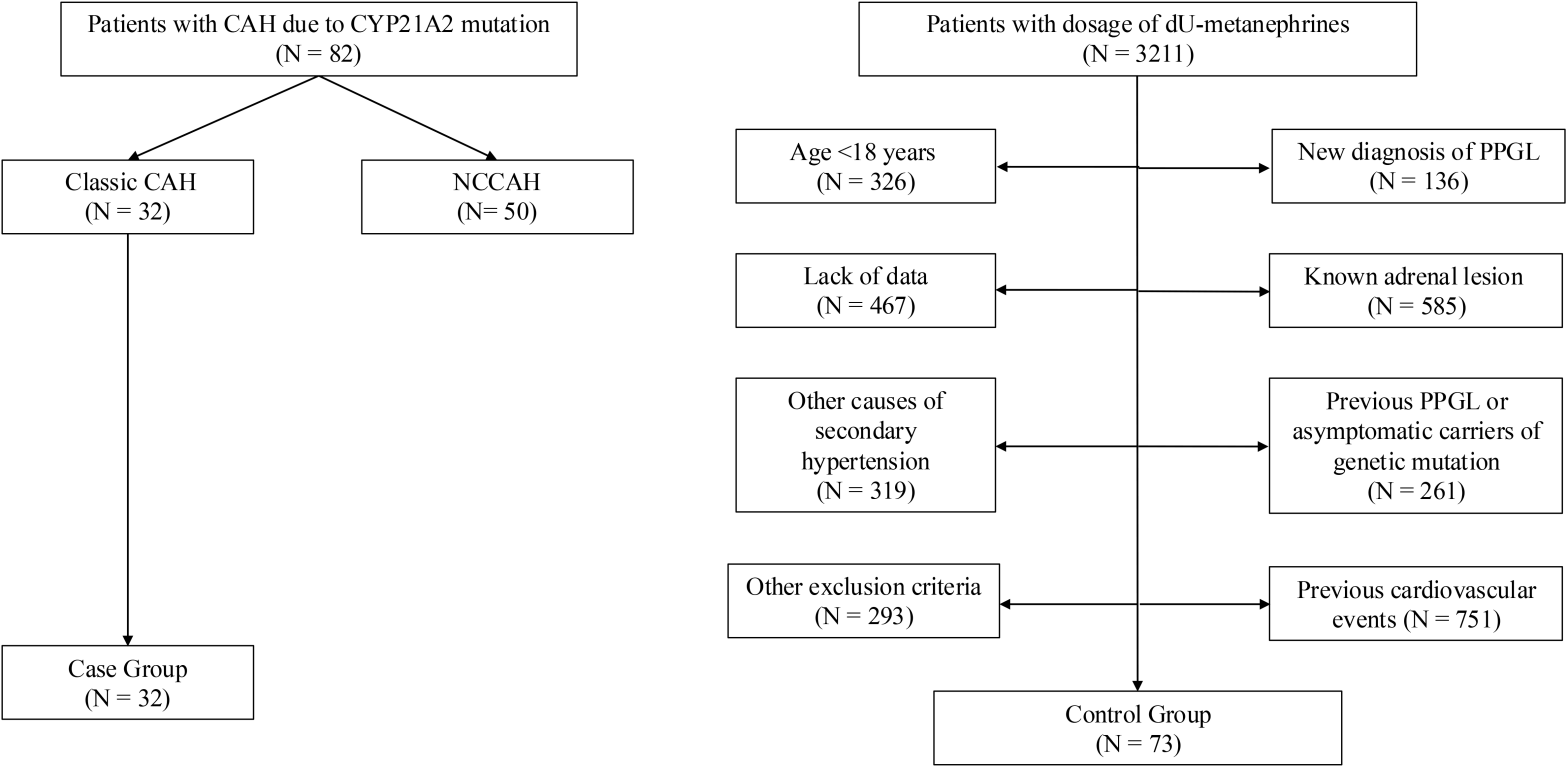
Study flow-chart.

Enrolled population was composed of 105 subjects, 32 classic CAH patients and 73 controls (**Figure 1**). Out of the 32 CAH patients: 21 were females (66%) and 11 were males (34%), their median age was 25 (19.5–29.5) years. Most patients were diagnosed at birth, with a median disease duration of 24 (19–29) years (**Tables 1**, **2**).

**Table 1:** Univariate analysis: differences between controls and CAH groups, population (table above) and instrumental features (table below).

	Total	Controls	CAH	p-value
N	105 (100.0%)	73 (69.5%)	32 (30.5%)	
**Age (years)**	30.0 (24.0-36.0)	33.0 (26.0-37.0)	25.5 (19.5- 29.5)	0.035
**Male Sex**	30.5%	28.8%	34.4%	0.566
**Weight (Kg)**	70.0 (58.0-84.5)	72.5 (64.0-90.0)	62.0 (56.0-73.5)	0.006
**BMI (kg/m^2^)**	24.9 (21.5-29.3)	25.5 (22.5-29.4)	23.8 (20.7-27.5)	0.457
**Office SBP (mmHg)**	130.0 (120.0-130.0)	130.0 (125.0-130.0)	120.0 (110.0-130.0)	<0.001
**Office DBP (mmHg)**	80.0 (72.5-85.0)	80.0 (70.0-85.0)	80.0 (73.0-80.0)	0.122
**Smoking Habit**	36.2%	46.6%	12.5%	<0.001
**Sodium (mmol/L)**	140.0 (138.0-141.0)	140.0 (138.0-142.0)	139.0 (138.0-141.0)	0.652
**Potassium (mmol/L)**	4.1 (3.8-4.4)	4.1 (3.7-4.3)	4.2 (4.0-4.5)	0.031
**Glucose (mg/dL)**	83.0 (75.0-90.5)	85.0 (76.5-94.0)	76.0 (72.5- 85.5)	0.007
**IFG**	4.8%	5.5%	3.1%	0.694
**Diabetes**	1.0%	1.4%	0.0%	0.694
**Total Cholesterol (mg/dL)**	183.0 (156.0-219.0)	192.0 (171.0-223.0)	161.0 (139.0-188.0)	<0.001
**HDL (mg/dL)**	49.0 (40.0-62.0)	47.5 (39.0-60.0)	56.0 (46.0-63.0)	0.033
**Triglycerides (mg/dL)**	95.5 (69.0-131.0)	100.0 (75.0-157.0)	75.0 (54.0-106.0)	0.025
**LDL (mg/dL)**	110.8 (90.0-141.0)	121.8 (97.6-143.2)	82.4 (73.0-110.0)	<0.001
**Creatinine (mg/dL)**	0.80 (0.66-0.92)	0.80 (0.67-0.91)	0.72 (0.65-0.92)	0.588
**eGFR (CKD-EPI) (mL/min/1.73m^2^)**	97.9 (81.0-114.4)	96.4 (80.0-113.2)	100.0 (81.7-120.0)	0.549
ECG				
**HR (bpm)**	68.0 (60.0-79.0)	68.0 (60.0-76.0)	71.0 (65.5-79.5)	0.371
**QTc (ms)**	400 (386-430)	410 (390-430)	395 (378-401)	0.006
**RR (ms)**	880 (760-1000)	900 (790-1000)	849 (740-915)	0.045
**PQ (ms)**	160 (138-174)	160 (140-180)	155 (130-163)	0.093
**QRS (ms)**	82 (80-100)	80 (80-100)	90 (82-105)	0.004
ABPM				
**24-h mSBP (mmHg)**	124.0 (114.2-134.7)	124.1 (115.7- 135.0)	118.6 (108.8-132.9)	0.165
**24-h mDBP (mmHg)**	75.9 (69.1-83.3)	76.8 (71.8-85.3)	73.4 (67.0-77.6)	0.025
**24-h mHR (bpm)**	78.2 (72.1-83.3)	78.2 (71.5-83.1)	79.2 (75.2-84.2)	0.126
**24-h HRV (bpm)**	12.3 (10.0-16.0)	11.5 (9.6-14.9)	14.2 (12.3-17.5)	0.007
**24-h mPP (mmHg)**	47.6 (39.9-52.0)	47.6 (41.0-52.0)	45.2 (39.3-51.8)	0.090
**Daytime mSBP (mmHg)**	130.5 (118.3-142.0)	130.5 (122.2-142.8)	123.4 (115.6-137.1)	0.147
**Daytime mDBP (mmHg)**	79.8 (74.7-88.7)	80.5 (75.0-90.4)	78.3 (68.3-81.4)	0.020
**Daytime mHR (bpm)**	82.6 (74.7-88.8)	81.5 (74.0-88.8)	83.0 (80.8-88.9)	0.108
**Daytime HRV (bpm)**	11.2 (9.2-14.5)	10.7 (8.5-13.9)	13.2 (11.3-15.6)	0.002
**Daytime mPP (mmHg)**	49.2 (42.1-53.7)	49.2 (41.1-53.7)	48.9 (42.6-53.4)	0.859
**Nighttime mSBP (mmHg)**	109.0 (100.7-120.5)	109.7 (103.8-121.8)	104.4 (92.4-116.9)	0.058
**Nighttime mDBP (mmHg)**	65.9 (58.2-71.9)	67.3 (59.3-73.6)	60.9 (55.2-65.4)	0.007
**Nighttime mHR (bpm)**	66.8 (61.8-72.0)	68.2 (61.8-72.3)	65.6 (62.3-71.4)	0.555
**Nighttime HRV (bpm)**	6.2 (4.6-7.9)	6.2 (4.4-7.2)	6.6 (5.1-9.6)	0.090
**Nighttime mPP (mmHg)**	43.9 (38.1-49.7)	43.6 (38.1-50.4)	44.1 (37.7-48.8)	0.718
**AASI**	0.385 (0.285-0.460)	0.350 (0.260-0.440)	0.430 (0.380-0.550)	0.006
Dipping				
***Reverse***	1.0%	0.0%	4.3%	0.220
***Reduced***	24.0%	24.7%	21.7%	
***Normal***	52.1%	54.8%	43.5%	
***Extreme***	22.9%	20.5%	30.4%	

AASI, Ambulatory Arterial Stiffness Index; ABPM, Ambulatory Blood Pressure Monitoring; BMI, Body Mass Index; CAH, Congenital Adrenal Hyperplasia; DBP, Diastolic Blood Pressure; ECG, Electrocardiogram; eGFR, estimated Glomerular Filtration Rate; HDL, High Density Lipoprotein; HR, Heart Rate; HRV, Heart Rate Variability; IFG, Impaired Fasting Glucose; LDL, Low Density Lipoprotein; mDBP, mean Diastolic Blood Pressure; mDP, mean Differential Blood Pressure; mHR, mean Heart Rate; mSBP, mean Systolic Blood Pressure; SBP, Systolic Blood Pressure.

Values are expressed as median (25th–75th percentile) or percent values.

**Table 2:** Data of patients with CAH.

CAH	
N	32
SW forms	28 (87.5%)
Years from diagnosis	24.0 (19.0- 29.0)
HC equivalent dose (mg/day)	20.0 (20.0-25.0)
FLU dose (SW only) (µg/day)	100.0 (67.5-100.0)
ACTH (pg/mL)	75.6 (22.5-199.0)
17OHP (ng/mL)	20.5 (2.3-73.0)
Androstenedione (ng/mL)	1.61 (0.50-4.90)
DHEAS (µg/L)	267.0 (94.0-456.0)
T (male only) (ng/mL)	3.44 (2.73-5.95)
PG (female only) (ng/mL)	4.11 (0.80-9.91)
HC	68.8%
DR-HC	15.6%
DEX+HC	15.6%
Osteopenia	33.3%
Osteoporosis	9.5%
TARTS	36.4%
PCOM	23.5%

17OHP, 17-Hydroxyprogesterone; ACTH, Adrenocorticotropic Hormone; CAH, Congenital Adrenal Hyperplasia; DEX, Dexamethasone; DHEAS, Dehydroepiandrosterone-sulphate; DR-HC, Double Release-Hydrocortisone; HC, Hydrocortisone; FLU, Fludrocortisone; PCOM, Polycystic Ovarian Morphology; PG, Progesterone; SW, Salt-Wasting; T, Testosterone; TARTS, Testicular Adrenal Rest Tumors.

Values are expressed as median (25th–75th percentile) or percent values.

Regarding GCs therapy, most CAH patients (22 cases, 68.8%) were treated with hydrocortisone (HC), while 5 patients (15.6%) received dual-release HC (DR-HC) and another 5 patients (15.6%) were on a combination therapy (HC or cortisone acetate plus dexamethasone). Salt-wasting forms (28 cases, 87.5%) were treated with fludrocortisone. The median daily HC equivalent dose was 20.0 (20.0–25.0) mg, and the median daily dose of fludrocortisone was 100 (67.5–100) µg.

When compared to the control group, CAH patients were younger (25, 19.5–29.5 vs 33, 26–37 years, p=0.035) and had a significantly lower body weight (62, 56–73.5 vs. 72.5, 64–90 kg; p=0.006). Office systolic blood pressure (SBP) (130, 125–130 vs 120, 110–130 mmHg; p<0.001) and smoking prevalence (46.6% vs. 12.5%; p<0.001) were significantly higher among controls, compared to CAH patients. CAH patients had significantly higher potassium levels (4.2, 4.0–4.5 vs. 4.1, 3.7–4.3 mmol/L; p=0.031) and lower fasting blood glucose (76, 72.5–85.5 vs 85, 76.5–94 mg/dL; p=0.007), compared to controls. However, no differences were found in the prevalence of impaired fasting glucose or diabetes mellitus. CAH patients’ lipid profile differed significantly from the control group, with a lower total cholesterol (161, 139–188 vs 192, 171–223 mg/dL, p<0.001); higher HDL cholesterol (56, 46–63 vs 47.5, 39-60; p=0.033), lower triglycerides (75, 54–106 vs 100, 75-157; p=0.025) and lower LDL-cholesterol (82.4, 73–110 vs 121.8, 97.6-143.2; p<0.001). No differences in terms of kidney function were found between the groups.

Patients with CAH exhibited a shorter QTc (395, 378–401 vs 410, 390–430 ms, p<0.006) and RR interval (849, 740–915 vs 900, 790–1000 ms, p=0.045) and a longer QRS complex (90, 82–105 vs 80, 80–100 ms; p=0.004) (**Table 1**). The mean diastolic blood pressure (DBP) at ABPM, was significantly lower in CAH patients in the 24-hour (73.3, 66.9–77 vs 76.7, 71.7-82.2 mmHg; p=0.025), as well as taking into consideration daytime (78.3, 68.3-81.4 vs 80.5, 74.9-90.3 mmHg; p=0.020) and nighttime (60.9, 55.1-65.4 vs 67.2, 59.2-73.5 mmHg; p=0.007). Heart rate variability (HRV), measured through ABPM, showed differences between the groups with a higher variability in CAH patients during the 24-hour period (14.2, 12.3-17.5 vs 11.4, 9.5-14.9 bpm; p=0.007), as well as in daily hours (13.1, 11.3-15.5 vs 10.7, 8.5-13.9 bpm; p=0.002), in comparison with the control group. No differences were found in dipping profile among the two groups.

The Ambulatory Arterial Stiffness Index (AASI), evaluated through ABPM, was significantly increased in patients with CAH compared to controls (0.430, 0.380-0.550 vs 0.350, 0.260-0.440; p=0.006).

### Multiple linear regression and propensity score matched analyses

Multiple linear regression showed that CAH diagnosis (exponentiated coefficient [EC] 1.131, 95% CI 1.061-1.205; p<0.001) was positively associated with AASI, correcting for age (EC1.004, 95% CI 1.000-1.008; p=0.034), male sex, BMI (EC1.008, 95% CI 1.003-1.011, p<0.001), glucose alterations, LDL-cholesterol and smoking habit (**Table 3** – Model A). When the regression was calculated considering only the CAH group, a significant association was found between 17α-hydroxyprogesterone (17αOHP) levels (EC 1.001, 95% CI 1.000-1.0002; p=0.049) and AASI, correcting for the same covariates (**Table 3** – Model B).

**Table 3:** Multiple linear regression analysis for Ambulatory Arterial Stiffness Index in the whole population (Model A) and in the CAH subgroup (Model B).

Model A	Covariates	Coefficient	CI 95%	p-value
AASI				
	CAH diagnosis	1.131	1.061-1.205	<0.001
	Age	1.004	1.000-1.008	0.034
	Male Sex	1.024	0.967-1.083	0.415
	BMI	1.008	1.003-1.012	<0.001
	Diabetes/Prediabetes	1.039	0.954-1.131	0.375
	LDL-cholesterol	1.000	0.999-1.001	0.696
	Smoking Habit	1.042	0.984-1.102	0.155

Model B	Covariates	Coefficient	CI 95%	p-value
AASI				
	17αOHP	1.001	1.000-1.002	0.049
	Age	1.005	1.000-1.010	0.071
	Male Sex	1.079	0.969-1.202	0.149
	BMI	1.007	1.000-1.015	0.054
	Diabetes/Prediabetes	0.935	0.750-1.166	0.524
	LDL-cholesterol	1.000	0.998-1.002	0.994
	Smoking Habit	1.066	0.930-1.222	0.331

17αOHP, 17α-Hydroxyprogesterone; AASI, Ambulatory Arterial Stiffness Index; BMI, Body Mass Index; CAH, Congenital Adrenal Hyperplasia; IFG, Impaired Fasting Glucose; LDL, Low Density Lipoprotein.

In another multiple linear regression model, the length of the CAH diagnosis (EC0.977, 95% CI 0.956-0.999; p=0.039) proved to be negatively associated with QTc interval at the ECG, when correcting for age, male sex, potassium levels, BMI, ABPM 24-hour mean SBP and smoking habit (**Table 4**, Model A). When the model was applied to CAH patients only, ACTH (EC0.999; 95% CI 0.999-1.000; p=0.021) seems to be related to a shorter QTc, when normalizing for age (EC0.998, CI 95% 0.996-0.999; p=0.012), androstenedione levels, BMI, ABPM-24-hour mean SBP and smoking habit (**Table 4**, Model B).

**Table 4:** Multiple linear regression analysis for QTc interval in the whole population (Model A) and in the CAH subgroup (Model A).

Model A	Covariates	Coefficient	CI 95%	p-value
QTc				
	CAH diagnosis	0.977	0.956-0.999	0.039
	Age	1.000	0.998-1.001	0.766
	Male Sex	0.998	0.977-1.019	0.847
	Potassium	1.017	0.994-1.040	0.146
	BMI	1.000	0.998-1.000	0.446
	ABPM mSBP	1.000	1.000-1.001	0.560
	Smoking Habit	1.019	0.999-1.038	0.058

Model B	Covariates	Coefficient	CI 95%	p-value
QTc				
	ACTH	0.999	0.999-1.000	0.021
	A4	1.001	0.995-1.006	0.672
	Age	0.998	0.996-0.999	0.012
	BMI	0.998	0.996-1.000	0.095
	ABPM mSBP	1.000	0.999-1.001	0.287
	Smoking Habit	1.053	0.995-1.113	0.068

A4, Androstenedione; ABPM, Ambulatory Blood Pressure Monitoring; ACTH, Adrenocorticotropic Hormone; BMI, Body Mass Index; CAH, Congenital Adrenal Hyperplasia; ECG, Electrocardiogram; mSBP, mean Systolic Blood Pressure.

When the two subgroups were studied through the propensity score matched analysis with a 1:2 matching ratio, based on the same regression models, CAH diagnosis confirmed a significant positive association with increased AASI (EC0.09, 95% CI 0.069-0.117; p<0.001) and a negative association with the QTc interval length (EC-0.028, 95% CI -0.046 - -0.009; p=0.004) (**Table 5**).

**Table 5:** Propensity score matched analyses for Ambulatory Arterial Stiffness Index and QTc interval between CAH patients and controls with a 1:2 matching.

	Coefficient	95% CI	p-value
**AASI**	0.093	0.069 - 0.117	<0.001
**ECG-QTc**	-0.028	-0.046 - -0.009	0.004

ABPM, Ambulatory Blood Pressure Monitoring, CI, Confidence Interval, ECG, Electrocardiogram.

## Discussion

The results of the present study show that adult patients affected by classic CAH due to 21β-hydroxylase deficiency present an increased arterial stiffness, assessed by AASI, and a shorter QT interval, when compared with controls.

We included a sample of adult classic CAH patients and compared its metabolic and cardiovascular features with a control group of young patients (<40 years) with the suspicion of pheochromocytoma/paraganglioma, in whom a chromaffin tumor was excluded. Despite a poorer metabolic and cardiovascular profile of the control group, the AASI appeared to be higher in CAH patients when compared to controls. The multiple regression model confirmed this association when normalizing for variables such as age, sex, BMI, the presence of glucose metabolism alterations, LDL-cholesterol levels and smoking habit; with CAH patients showing a higher risk of having an increased AASI, furthermore confirmed by matching the two groups through a propensity score-matched analysis, based on the same regression models. When replicated among the CAH group, the multiple regression model showed a positive relationship between 17αOHP levels and AASI. Although previous studies on AASI as a cardiovascular parameter in adult CAH patients are lacking, a study conducted on pediatric CAH reported an increased AASI in Del/Del and Del/I2G genotypes and found a relationship with urinary cortisol, cortisol area under the curve and cortisol after the first dose of HC (16). Several studies have found a positive relationship between CAH and an increased thickness of the carotid-artery intima media (17), with a significant correlation between a worse vascular system health and 17αOHP levels (18), despite HC equivalent doses, highlighting the possible role of androgen excess on carotid atherosclerosis. Other cardiac alterations, such as prolonged isovolumetric relaxation and mitral deceleration times have been reported in CAH adolescents in relationship with hydrocortisone exposure and elevated testosterone levels (19).

Heart-rate variability (HRV) was also significantly lower in our control group compared to CAH patients, in addition to the worse conventional risk factors presented, strengthening our findings. HRV is a robust prognostic indicator of cardiovascular health, reflecting autonomic balance and cardiac–brain communication: higher HRV denotes increased parasympathetic tone and superior cardiac fitness, whereas lower HRV is associated with elevated cardiovascular risk (20–22). The subgroup of CAH patients still showed markedly increased arterial stiffness despite better cardiovascular health markers. This finding underscores an intrinsic vascular dysfunction in CAH that appears independent of traditional metabolic and lifestyle risk markers and could be linked to circulating androgen levels.

Patients with CAH present a duration of the QTc interval that could be modulated by the combination of excess hormones expressed. In the present study, we found a significant effect of CAH on QTc values, with smaller intervals when compared with the control group even after a propensity score matched analysis, further affirming the link between CAH and modulation of the QTc interval.

When applying the same model on the CAH group only, ACTH seems to be negatively associated with QTc interval length. A previous study (23) found an association between women with CAH and a shorter QTc interval, linking this alteration to lower FSH levels and a higher progesterone/estradiol ratio. Although gonadotropins were not measured in the present study, a higher ACTH is associated with an increased secretion of adrenal steroids with a potential increased impact on gonadotropins, which could lead to an impaired LH pulsatility and to lower FSH levels as a feedback mechanism (24), partially explaining the similar features found in our study.

Despite the use of a rigorous study protocol, our study shows some limitations. The sample size and the cross-sectional nature of the study may limit the generalizability of results, moreover the impact of different GCs treatment approaches on cardiometabolic features have not been studied due to the small sample size. It will be essential to extend the research to a larger sample and a multicenter context. The analysis of the link between hormone levels, such as ACTH and sexual hormones and cardiovascular parameters may provide further insights into the management of CAH.

In conclusion, the present study has provided new insights into the understanding of cardiometabolic pathophysiology of classic CAH due to 21β-hydroxylase deficiency. Significant differences were found in parameters of arterial stiffness (AASI) and the QTc interval, and androgen and ACTH excess could lead to these cardiovascular alterations. Further prospective studies are needed to confirm our findings.

## Data Availability

The datasets generated and/or analyzed during the current study are available from the corresponding author upon reasonable request.
